# Intergenerational Strain and Subjective Well-being: The Role of Leisure Activity Engagement

**DOI:** 10.1093/geroni/igab046.2909

**Published:** 2021-12-17

**Authors:** Amanda Collins, Jeffrey Stokes, Elizabeth Dugan

**Affiliations:** 1 University of Massachusetts Boston, Boston, Massachusetts, United States; 2 UMass Boston, Boston, Massachusetts, United States

## Abstract

Family strain is associated with higher numbers of depressive symptoms and lower levels of life satisfaction. Leisure activities are observed to buffer the negative effects of family strain among younger adults, however, this phenomenon is understudied among older adults. This study examines the relationship between intergenerational strain and depressive symptoms and life satisfaction among persons aged 50 and older. The study also examines the moderating effects of gender and leisure activities. The analysis uses the Health and Retirement Study to addresses these questions. The results suggest that intergenerational strain (p=.000) and being female (p.=000), are associated with more depressive symptoms, while engagement in social leisure activities (p.=04) is associated with fewer. Intergenerational strain (p=.000) and being female (p=.03) are associated with lower levels of life satisfaction, while engagement in solitary (p=.000) and social leisure activities (p=.000) are associated with higher levels. Results from moderation models suggest that as intergenerational strain increases, women have lower life satisfaction and more depressive symptoms as compared to men (p=.000). Also, the association between intergenerational strain and life satisfaction is reduced among respondents who engaged in leisure activities (p=.002-social and p=.000-solitary). Further, the positive relationship between intergeneration strain and depressive symptoms is lower for persons who engage in leisure activities (p=.027-solitary and p=.013-social). Finally, women who engage in social and solitary leisure activities have fewer depressive symptoms than men (p=.037). The study findings imply that the subjective well-being of older persons may be improved in terms of intergeneration strain if they engage in leisure activities.

